# Difficult-to-detect carbapenem-resistant IMP13-producing *P. aeruginosa*: experience feedback concerning a cluster of urinary tract infections at a surgical clinic in France

**DOI:** 10.1186/2047-2994-2-12

**Published:** 2013-04-04

**Authors:** Odile Milan, Laurent Debroize, Xavier Bertrand, Patrick Plesiat, Anne-Sophie Valentin, Roland Quentin, Nathalie Van der Mee-Marquet

**Affiliations:** 1 Clinique Notre dame de Bon Secours, Chartres, France; 2 Laboratoire d’Analyses Médicales, Luisant, France; 3 Service d’Hygiène, Centre Hospitalier Universitaire, Besançon, France; 4 UMR 6249 Chrono-environnement, Université de Franche-Comté, Besançon, France; 5 Centre National de référence, Centre Hospitalier Universitaire, Université de Franche-Comté, Besançon, France; 6 Service de Bactériologie et Hygiène, Tours, France; 7 Réseau des Hygiénistes du Centre, Hôpital Trousseau, Centre Hospitalier Universitaire, Tours, France

## Abstract

**Background:**

We report a carbapenem-resistant *P. aeruginosa* clone responsible for a cluster of urinary tract infections in elderly surgery patients, diagnosed during a three-month period in a 59-bed surgical clinic.

**Findings:**

The clonal nature of the cluster was established by molecular study of the *P. aeruginosa* isolates (PFGE and MLST). Despite an MIC of imipenem in the susceptibility range for two isolates, all were metallo-β-lactamase-producers (IMP13-type, clone ST621). We conducted a review of the medical and surgical procedures. We tested water delivered into the clinic and urological devices for the presence of the epidemic strain. The hygiene nurse observed hygiene practices. A week after the implementation of barrier precautions around the fourth infected patient, we studied the extent to which the patients hospitalised were colonised to assess whether the spread of the epidemic strain had been controlled.

**Conclusions:**

1/ Our findings indicate the difficulties in the detection of the metallo-β-lactamase in this clone, that resulted in the alert being delayed. 2/ Unlike most investigations of UTI outbreaks described in urology wards, we did not detect any contaminated urological devices or water colonisation. 3/ Consistent with outbreaks involving the IMP-13 clone in critical care units, the observation of inadequate application of standard precautions argued for patient-to-patient transmission during urinary management of the urology patients. 4/ The implementation of barrier precautions around infected patients resulted in control of the spread of the epidemic clone. This report serves as an alert concerning a difficult-to-detect multidrug-resistant *P. aeruginosa* clone in elderly urology patients.

## Introduction

IMP-type enzymes, the first acquired metallo-β-lactamases (MBLs) to be detected in Gram-negative pathogens in the early 1990s, remain among the most prevalent and widely distributed MBLs. The *P. aeruginosa* clone ST621, producing IMP-13 [1], has been responsible for outbreaks in Italian critical care settings, and has recently been identified in other European countries and in South America [1-3].

Patient-to-patient transmission of *Pseudomonas aeruginosa* is frequent in high-risk patients. Numerous outbreaks of multi-drug resistant *P. aeruginosa* have been described in neonatal and adult intensive care units, burns units, oncohaematology units and transplantation units [4]. Outside critical care settings, nosocomial outbreaks of *P. aeruginosa* are mostly associated with contaminated water supplies or inadequately disinfected medical or surgical devices [5]. In urology wards, *P. aeruginosa* has been found to be associated with urinary tract infection (UTI) outbreaks linked to contaminated urodynamic systems, cystoscopes or urometers [6], or tap water colonisation [7].

We report a cluster of UTIs associated with a carbapenem-resistant IMP13-producing *P. aeruginosa* strain in a French small surgical clinic. This report serves as an alert concerning a difficult-to-detect and multidrug-resistant *P. aeruginosa* clone in elderly urology patients.

## Methods

The *P. aeruginosa* isolates from the UTI cases were identified with Vitek 2 Gram-Negative Identification Cards (bioMerieux, France). Antibiotic susceptibility was tested by an agar disk diffusion method. The combined meropenem +/− dipicolinic acid disk test (Rosco, France) was used for the phenotypic detection of MBL in isolates displaying decreased susceptibility to imipenem according to CLSI and EUCAST breakpoints (MIC > 4 mg/L). The metallo-β-lactamase gene, *bla*IMP-13, was identified in the epidemic strain as previously described [2]. The isolates were also characterised by O-serotyping, pulsed-field gel electrophoresis (PFGE) and MLST as previously described [8]. We also studied three unrelated IMP-13-producing *P. aeruginosa* recently isolated in distant French regions (named R1 of ST 621, R2 of ST 308 and R3 of ST111).

During the outbreak investigation, we tested water delivered into the clinic and urological devices for the presence of the epidemic strain. Environmental samples (n=26) were taken from water fittings in each medical and surgical unit. Samples of 200 mL of cold water taken directly from the tap immediately after activating were filtered and the filters cultured on plates containing cetrimide medium. Environmental samples were also taken from the four cystoscopes of the clinic. Plates were incubated at 37°C for 48 hours, and all bacterial colonies likely to be *P. aeruginosa* were identified and studied as described above.

The hygiene nurse observed hygiene practices (materials and techniques used) in the clinic, to assess if the standard precautions (for all patients) and the barrier precautions (around IMP-13-producing *P. aeruginosa*-infected patients) were being followed by healthcare workers.

A week after the implementation of barrier precautions around the fourth infected patient, we studied the extent to which the patients hospitalised in the clinic were colonised with the epidemic strain to assess if its spread had been controlled. Rectal swabs were taken from each patient for screening. Swabs were immediately suspended in 0.5 mL of sterile water and 0.1 mL of the suspension was streaked on a plate containing cetrimide medium.

This study was run in accordance with the French Healthcare recommendations for the prevention of infection. Ethical approval was obtained at the national level from the Réseau Alerte Investigation Surveillance des Infections Nosocomiales (RAISIN). The study was managed jointly with the director of the clinic, the hygiene nurse, surgeons and physicians responsible for caring for the patients, and the regional infection-control practitioner. Patients and their relatives were enrolled after an individual interview for consent to allow access to medical records and to culture a faecal sample.

## Results

### The four clinical cases of UTI

The first case involved a 74-y-old man diagnosed on the 28^th^ of October with a multidrug-resistant imipenem-intermediate *P. aeruginosa* UTI, 45 days after prostatectomy, and following imipenem treatment administered for multidrug-resistant imipenem-susceptible *P. aeruginosa* UTI identified one week earlier*.* The second case was a 90-y-old man diagnosed on the 19^th^ of December with a multidrug-resistant imipenem-susceptible *P. aeruginosa* UTI, 15 days after nephrostomy. Patient 3 (80 y) was diagnosed on the 6^th^ of January with a multidrug-resistant imipenem-susceptible *P. aeruginosa* UTI, one day after cystography and during a period when he was undergoing numerous urological surgical interventions. The fourth patient was a woman (89 y) diagnosed on the 16^th^ of January with a multidrug-resistant imipenem-intermediate *P. aeruginosa* UTI, one month after nephrostomy. In all cases, UTIs were defined on the basis of clinical signs of infection and biological criteria (significant leukocyturia and bacteriuria).

### Detection of the IMP-13-producing P. aeruginosa

**Figure 1:**
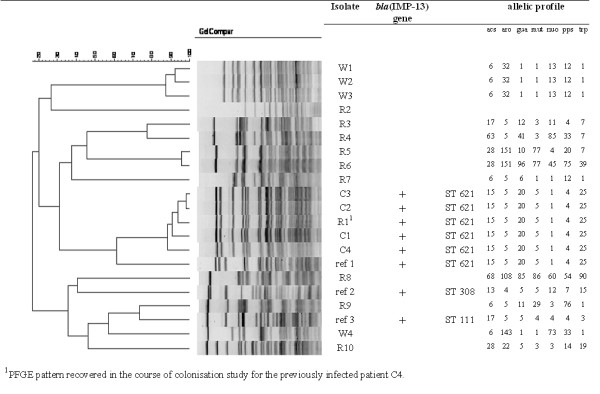
**Pulsed-field gel electrophoresis patterns of the four UTI-associated IMP-13 *P. aeruginosa *isolates (C1-C4), the ten colonising isolates recovered from carriers (R1-R10), the four environmental isolates recovered from water samples (W1-W4), and three epidemiologically unrelated IMP-13 *P. aeruginosa *reference isolates (ref. 1–3). **^1^PFGE pattern recovered in the course of colonisation study for the previously infected patient C4.

The MICs of imipenem were 8 mg/L for isolates from patients 1 and 4. The *P. aeruginosa* isolates associated with these UTIs exhibited a high level of resistance to ceftazidime (>256 mg/L). Following the French Healthcare recommendations, the two isolates showing a decreased susceptibility to imipenem (MIC=8 mg/L) were screened for MBL. Both isolates were found to be MBL-producers. The clonal nature of the two isolates was established from the PFGE patterns of the isolates. As a consequence of these findings, a retrospective study of *P. aeruginosa* UTIs was conducted. Two UTIs associated with ceftazidime-resistant *P. aeruginosa* isolates were identified in patients 2 and 3; the MICs of imipenem were 4 mg/L for both isolates. We studied the two ceftazidime-resistant imipenem-susceptible *P. aeruginosa* isolates recovered from patients 2 and 3, even though their susceptibility to imipenem was not below the threshold value. These isolates were also MBL-producers. The clonal nature of the UTI cluster was then established from the PFGE patterns of the four isolates (Figure 1). All belonged to serogroup O:4, and were identified as being IMP13-type and members of clone ST621, also called the Italian clone. These investigations therefore established a cluster of four UTIs associated with an IMP-13-producing epidemic strain, diagnosed during a three-month period in a 59-bed surgical clinic in Chartres, France.

### Review of procedures and techniques, and analysis of environmental samples and hygiene practices

A review of the medical and surgical procedures, and other techniques performed by the medical staff, did not reveal any factor common to the infected patients. *P. aeruginosa* isolates were recovered from four water samples collected from water fittings in the urological surgical unit, but none of these isolates exhibited high levels of resistance to any of the antibiotics tested. In addition, PFGE and MLST demonstrated that these environmental isolates were genetically distant from the epidemic IMP-13 strain (Figure 1). None of the environmental samples from the cystoscopes was positive for *P. aeruginosa*. Observations by the hygiene nurse revealed that the barrier precautions around IMP-13-producing *P. aeruginosa*-infected patient 4 were being followed by healthcare workers. By contrast, the application of standard precautions was not optimal, as hand hygiene practices were inadequate, especially for urinary management and/or the manipulation of urinary catheters, and before cystography. Overall, these observations suggest that transmission between patients was not likely to have been associated with surgery, invasive acts or contaminated water supplies, but with urinary managment.

### Nosocomial acquisition of the IMP-13 P. aeruginosa after implementation of barrier precautions

On the 23^th^ of January, all patients hospitalised in the clinic, were screened for the epidemic strain. Nine of the 55 patients (16%), all urology patients, were positive for *P. aeruginosa* carriage. PFGE and MLST showed genetically diverse colonising isolates and that none presented the IMP13-type (Figure 1). Note that the colonising isolate (R1) of ST 621 and presenting the PFGE IMP13 pattern, was recovered from patient 4, who was still hospitalised at the time of the colonisation study. In addition, no epidemic strain was recovered from any patient at the clinic during the following six months, suggesting that the spread of the epidemic isolate in the clinic had been controlled.

## Discussion

Our results document the spread of the IMP13-producing clone in a small private surgical clinic, rather than in an intensive care unit of a university hospital as previously described for this clone.

For two of the four isolates, the MIC of carbapenem drugs was in the susceptibility range. Consequently, the detection of the cluster of UTIs was delayed for several weeks. Our findings confirm the previously described heterogeneous carbapenem-resistance phenotype of the IMP13-producing clone [9] and suggest that the spread of the clone may be, at least partially, facilitated by difficulties in its detection.

Despite extensive investigations prompted by this cluster of UTIs associated with the IMP-13 *P. aeruginosa*, no evidence was found for the involvement of the water supplies or contaminated medical or surgical devices. It is possible that the delay between the time of contamination of the patients and our investigations hindered the identification of a contaminated source. However, in all but one case, the time separating surgery and invasive acts from the onset of clinical signs of UTI was long. These observations do not argue for contamination of the patients during surgery or invasive acts.

By contrast, the temporal superposition of hospitalisation for the infected patients, and the observation of insufficiently strict application of hygiene practices in the urology unit and before cystography suggest that the UTI cases may have resulted from cross-transmission during post-surgery hospitalisation. The rate of faecal carriage of *P. aeruginosa* among elderly urology patients is frequently high, confirming the ability of this bacterium to colonise the urinary tract of such patients [10]. In view of the frequent and invasive nature of urinary care acts following surgery, and the well-described epidemic potential of the IMP-13 clone [1,3,11], we suggest that elderly urology patients should be considered to be at high risk of patient-to-patient cross-transmission of IMP-13-producing *P. aeruginosa*.

Concordant with MLST results, the recent sequence analysis of the *bla*IMP13 gene in numerous IMP-13-*P. aeruginosa* isolates from distant French regions showed that our epidemic isolates and the Italian clone have very similar characteristics [12]. Nevertheless, no link with countries experiencing IMP-13-*P. aeruginosa* outbreaks was identified in the index case history, and the origin of the IMP-13 clone involved in this outbreak remains unclear. Further studies are needed to improve our knowledge of the epidemiology of this highly resistant clone.

Because of the association between the use of broad-spectrum antibiotics and multidrug resistance, a campaign has been run to promote systematic microbiological documentation of UTI: this should favour the use of specific rather than broad-spectrum antibiotics. The early implementation of barrier precautions around infected patients successfully prevented the further spread of the carbapenem-resistant clone in the clinic. We suggest that, when facing a limited cluster of infections, such precautions may be sufficient for infection control, without the need for more extreme measures, such as cohorting, for example.

## Competing interest

The authors declare that they have no competing interest.

## Authors’contributions

NVDM conceived the study and wrote the manuscript. OD observed hygiene practices. LD isolated the epidemic strain and conducted the environmental study. Molecular characterization (PFGE) of the isolates was conducted by XB. ASV conducted MLST. XB, RQ and PP helped draft the manuscript. All authors read and approved the final manuscript.
